# Supplementary material for: Simulated Microgravity Effects on Human Adenocarcinoma Alveolar Epithelial Cells: Characterization of Morphological, Functional, and Epigenetic Parameters
Source: Int J Mol Sci. 2021 Jun 28;22(13):6951. doi: 10.3390/ijms22136951 (PMC8269359; doi:10.3390/ijms22136951)
Supplement: Supplementary file 1 [file ijms-22-06951-s001.zip › ijms-1255965-supplementary.pdf]

Suppl. Table S1. Down- and Up-regulated microRNA (selected >2-fold variation and $P < 0.05$) after microarray of A549 cells analysis of NG/MG pairs.

DOWN	UP
hsa-miR-1234-3p	hsa-let-7b-5p
hsa-miR-1260a	hsa-let-7d-5p
hsa-miR-151a-5p	hsa-miR-107
hsa-miR-151b	hsa-miR-10a-5p
hsa-miR-16-5p	hsa-miR-1202
hsa-miR-181a-5p	hsa-miR-1207-5p
hsa-miR-18a-5p	hsa-miR-125a-5p
hsa-miR-194-5p	hsa-miR-193b-3p
hsa-miR-20a-5p	hsa-miR-215-5p
hsa-miR-221-3p	hsa-miR-26a-5p
hsa-miR-22-3p	hsa-miR-29a-3p
hsa-miR-30c-5p	hsa-miR-3195
hsa-miR-3162-5p	hsa-miR-3198
hsa-miR-34a-5p	hsa-miR-320a
hsa-miR-3651	hsa-miR-320d
hsa-miR-374a-5p	hsa-miR-365a-3p
hsa-miR-4281	hsa-miR-3960
hsa-miR-4516	hsa-miR-425-5p
hsa-miR-4530	hsa-miR-4485-3p
hsa-miR-4687-3p	hsa-miR-4505
hsa-miR-5787	hsa-miR-4507
hsa-miR-6089	hsa-miR-4665-3p
hsa-miR-6090	hsa-miR-4672
hsa-miR-6124	hsa-miR-6088
hsa-miR-7641	hsa-miR-6734-5p
hsa-miR-940	hsa-miR-6749-5p
	hsa-miR-6800-5p
	hsa-miR-6821-5p
	hsa-miR-6869-5p
	hsa-miR-7107-5p
	hsa-miR-8069
	hsa-miR-96-5p
